# Differential long noncoding RNA/mRNA expression profiling and functional network analysis during osteogenic differentiation of human bone marrow mesenchymal stem cells

**DOI:** 10.1186/s13287-017-0485-6

**Published:** 2017-02-07

**Authors:** Wenyuan Zhang, Rui Dong, Shu Diao, Juan Du, Zhipeng Fan, Fu Wang

**Affiliations:** 1 Department of Oral Basic Science, School of Stomatology, Dalian Medical University, Liaoning, 116044 China; 2 Laboratory of Molecular Signaling and Stem Cells Therapy, Beijing Key Laboratory of Tooth Regeneration and Function Reconstruction, Capital Medical University School of Stomatology, Beijing, 100050 China

**Keywords:** MSCs, Gene expression, Long noncoding RNA, Osteogenic differentiation

## Abstract

**Background:**

Mesenchymal stem cells (MSCs) are the most promising cell types for bone regeneration and repair due to their osteogenic potential. MSC differentiation is precisely regulated and orchestrated by the mechanical and molecular signals from the extracellular environment, involving complex pathways regulated at both the transcriptional and post-transcriptional levels. However, the potential role of long noncoding RNA (lncRNA) in the osteogenic differentiation of human MSCs remains largely unclear.

**Methods:**

Here, we undertook the survey of differential coding and noncoding transcript expression profiling and functional network analysis during osteogenic differentiation of human bone marrow mesenchymal stem cells (BMSCs) using human whole transcriptome microarray. The key pathways, mRNAs, and lncRNAs controlling osteogenic differentiation of BMSCs were identified by further bioinformatic analysis. The role of lncRNA in the osteogenic differentiation of MSCs was verified by lncRNA overexpression or knockdown methods.

**Results:**

A total of 1269 coding transcripts with 648 genes significantly upregulated and 621 genes downregulated, and 1408 lncRNAs with 785 lncRNAs significantly upregulated and 623 lncRNAs downregulated were detected along with osteogenic differentiation. Bioinformatic analysis identified that several pathways may be associated with osteogenic differentiation potentials of BMSCs, such as the MAPK signaling pathway, the Jak-STAT signaling pathway, the Toll-like receptor signaling pathway, and the TGF-beta signaling pathway, etc. Bioinformatic analysis also revealed 13 core regulatory genes including seven mRNAs (GPX3, TLR2, BDKRB1, FBXO5, BRCA1, MAP3K8, and SCARB1), and six lncRNAs (XR_111050, NR_024031, FR374455, FR401275, FR406817, and FR148647). Based on the analysis, we identified one lncRNA, XR_111050, that could enhance the osteogenic differentiation potentials of MSCs.

**Conclusions:**

The potential regulatory mechanisms were identified using bioinformatic analyses. We further predicted the interactions of differentially expressed coding and noncoding genes, and identified core regulatory factors by co-expression networks during osteogenic differentiation of BMSCs. Our results could lead to a better understanding of the molecular mechanisms of genes and lncRNAs, and their cooperation underlying MSC osteogenic differentiation and bone formation. We identified that one lncRNA, XR_111050, could be a potential target for bone tissue engineering.

**Electronic supplementary material:**

The online version of this article (doi:10.1186/s13287-017-0485-6) contains supplementary material, which is available to authorized users.

## Background

Mesenchymal stem cells (MSCs) are the most promising cell types for bone regeneration and repair due to their osteogenic potential [1, 2]. Bone marrow mesenchymal stem cells (BMSCs) are considered the gold standard for use in bone tissue regeneration among MSCs, and they are an important source of multipotent progenitor cells with self-renewal capacity that can differentiate into osteoblasts, chondrocytes, and adipocytes with great potential for clinic applications [1, 3]. BMSC differentiation is precisely regulated and orchestrated by the mechanical and molecular signals from the extracellular environment, involving complex pathways regulated at both the transcriptional and post-transcriptional levels [4–6].

Long noncoding RNAs (lncRNAs) are a class of nonprotein coding genes. Recent advances in high-throughput technology and computational methods allow for an unprecedented analysis of such transcripts. Tens of thousands of lncRNAs transcribed from mammalian genomes have been identified [7–12]. Recent studies have shown the regulatory role and functional diversity of lncRNAs. Many functional lncRNAs show tissue-specific expression patterns and distinct subcellular localizations [9, 11, 13, 14]. Growing evidence suggests that lncRNAs are important modulators of gene expression via their interaction with DNA, RNA, or protein [15–25], while determining the function of individual lncRNAs remains a challenge. Some lncRNAs have been shown to regulate osteogenic differentiation of MSCs [26–31]. However, the particular functions of lncRNAs during osteogenic differentiation of human BMSCs still remain largely unclear.

A previous study found that osteogenic differentiation markers were enhanced and that the stemness marker NANOG was downregulated at day 7 of osteogenic differentiation induction in BMSCs, indicated that day 7 of the induction was the transit point of the onset of osteogenic differentiation in BMSCs [32]. In the present study, we aimed to investigate the key mRNAs and lncRNAs for the transit point of osteogenic differentiation in MSCs. Here, we performed microarray analysis to identify the differential coding and long noncoding transcript expression profiling between noninduced and day 7 osteogenic-differentiated human BMSCs. A total of 1269 coding transcripts and 1408 lncRNAs were identified with differential expression during osteogenic differentiation in BMSCs. We further predicted interactions between coding and noncoding genes, and identified core regulatory factors by bioinformatic analysis. Our results also highlight the significant involvement of one lncRNA, XR_111050, in the positive regulation of osteogenic differentiation of BMSCs.

## Results

### The profiling of differentially expressed mRNAs and lncRNAs during osteogenic differentiation of BMSCs

To identify the differentially expressed lncRNAs and mRNAs during osteogenic differentiation of MSCs, we first applied the RVM *t* test to filter the genes that were differentially expressed, and then the differentially expressed genes with 1.5-fold changes were selected according to the *p* value threshold false discovery rate (FDR) for subsequent analysis. A total of 1269 coding transcripts with differential expression were identified during osteogenic differentiation (*p* < 0.05, FDR < 0.05); of these, 648 were upregulated and 621 were downregulated in BMSCs at 7 days after osteogenic induction compared to the uninduced BMSCs (Additional file 1: Table S1). In addition, a total of 1408 lncRNAs were differentially expressed after osteogenic differentiation (*p* < 0.05, FDR < 0.05); of these, 785 were upregulated and 623 were downregulated in BMSCs at 7 days after osteogenic induction compared to the uninduced BMSCs (Additional file 2: Table S2).

To confirm the reliability of the microarray data, we randomly selected six differentially expressed mRNAs (FGF7, FOXO1, STAT2, TMEM156, FOXM1, and FOXC2), and nine differentially expressed lncRNAs (XR_111050, NR_003255, NR_027621, NR_002196, NR_045555, NR_024593, NR_034115, NR_034181, and NR_037595) to analyze their expression with real-time polymerase chain reaction (RT-PCR). The RT-PCR results confirmed that the expression levels of these randomly selected 15 genes were consistent with the microarray results (Table 1).

**Table 1:** The real-time polymerase chain reaction (RT-PCR) results showed differential mRNA and lncRNA expression levels during osteogenic differentiation of bone marrow mesenchymal stem cells

	RT-PCR results		Microarray results	
Gene symbol	0 days	7 days	7-day/0-day (fold-change)	Regulation
XR_111050	1.00 ± 0.063	32.76 ± 4.29**	14.13	Up
NR_045555	1.00 ± 0.11	4.08 ± 0.28**	5.14	Up
NR_002196	1.00 ± 0.089	10.08 ± 1.53**	4.7	Up
NR_024593	1.00 ± 0.24	2.16 ± 0.29**	4.23	Up
NR_003255	1.00 ± 0.11	3.78 ± 0.27**	2.68	Up
NR_027621	1.00 ± 0.081	2.86 ± 0.18**	1.88	Up
NR_034115	1.00 ± 0.19	0.20 ± 0.051**	0.27	Down
NR_037595	1.00 ± 0.27	0.57 ± 0.062**	0.32	Down
NR_034181	1.00 ± 0.085	0.39 ± 0.072**	0.49	Down
FGF7	1.00 ± 0.10	14.42 ± 1.27**	17.86	Up
FOXO1	1.00 ± 0.14	4.33 ± 0.46**	3.97	Up
STAT2	1.00 ± 0.19	2.61 ± 0.42**	2.8	Up
TMEM156	1.00 ± 0.12	0.25 ± 0.059**	0.084	Down
FOXM1	1.00 ± 0.21	0.17 ± 0.031**	0.27	Down
FOXC2	1.00 ± 0.19	0.59 ± 0.082**	0.41	Down

GAPDH was used as an internal control. The results represent mean ± standard deviation from three independent experiments (mean value of gene expression/GAPDH at day 0 was set as 1). Student’s *t* test was performed to determine statistical significance; **p < 0.01

### Bioinformatic analysis of microarray data during osteogenic differentiation of BMSCs

**Fig. 1:**
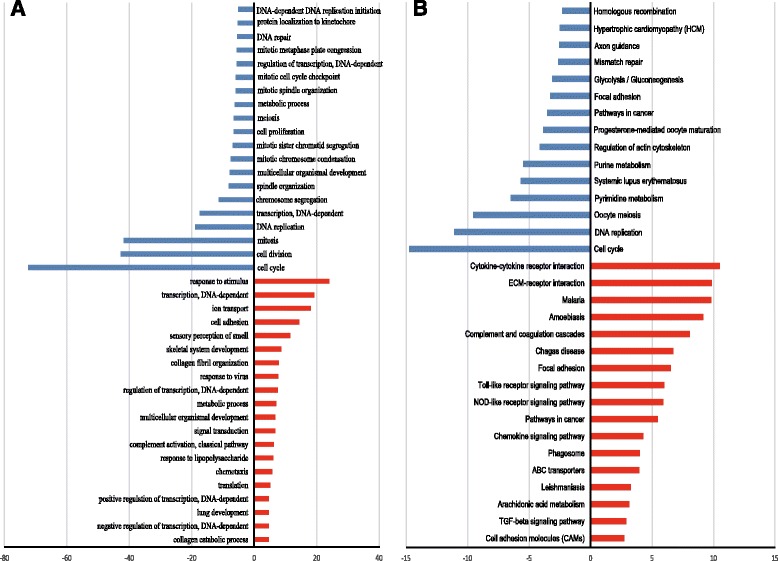
Significant gene ontology (GO) analyses and pathways of differentially expressed genes during osteogenic differentiation. **a** The significant GO of differentially expressed genes during osteogenic differentiation. **b** The significant pathways of differentially expressed genes during osteogenic differentiation. The *y* axis shows the GO or pathway category and the *x* axis shows the negative logarithm of the *p* value (–LgP). A larger –LgP indicated a smaller *p* value for the difference. *ECM* extracellular matrix, *TGF* transforming growth factor

To identify the key factors that controlled osteogenic differentiation of BMSCs, we performed gene ontology (GO) analysis and pathway analysis. First, GO analysis was performed to enrich the significant functions from the differentially expressed genes obtained during osteogenic differentiation of BMSCs. We obtained 171 GO functions according to *p* value and FDR (*p* < 0.05, FDR < 0.05) including 99 upregulated GO functions and 72 downregulated GO functions (Additional file 3: Table S3). The negative logarithm of the *p* value (–LgP) was used to represent the correlation between gene expression and the relevant biological process. Some important upregulated GO functions may be related to osteogenic differentiation, including response to stimulus, DNA-dependent transcription, ion transport, cell adhesion, and skeletal system development, and some important downregulated GO functions that were related to osteogenic differentiation were cell cycle, cell division, mitosis, DNA replication, and DNA-dependent transcription (Fig. 1a; Additional file 3: Table S3). We obtained 331 significantly upregulated genes and 297 significantly downregulated genes from enriched GO functions (*p* <0.01, FDR < 0.05).

We then enriched the significantly changed pathways that mediated the functions of the differentially expressed genes based on the KEGG database, and identified a total of 67 significant pathways related to differential gene expression which may play key roles during osteogenic differentiation of BMSCs (*p* < 0.05); of these, 37 pathways involved upregulated genes and 30 pathways involved downregulated genes (Fig. 1b; Additional file 4: Table S4). Some important pathways that may related to osteogenic differentiation were enriched from upregulated genes, including cytokine-cytokine receptor interaction, extracellular matrix (ECM)-receptor interaction, the Toll-like receptor signaling pathway, and the chemokine signaling pathway. Some important pathways may be related to osteogenic differentiation enriched from downregulated genes were cell cycle, DNA replication, pyrimidine metabolism, and purine metabolism (Fig. 1b; Additional file 4: Table S4).

**Fig. 2:**
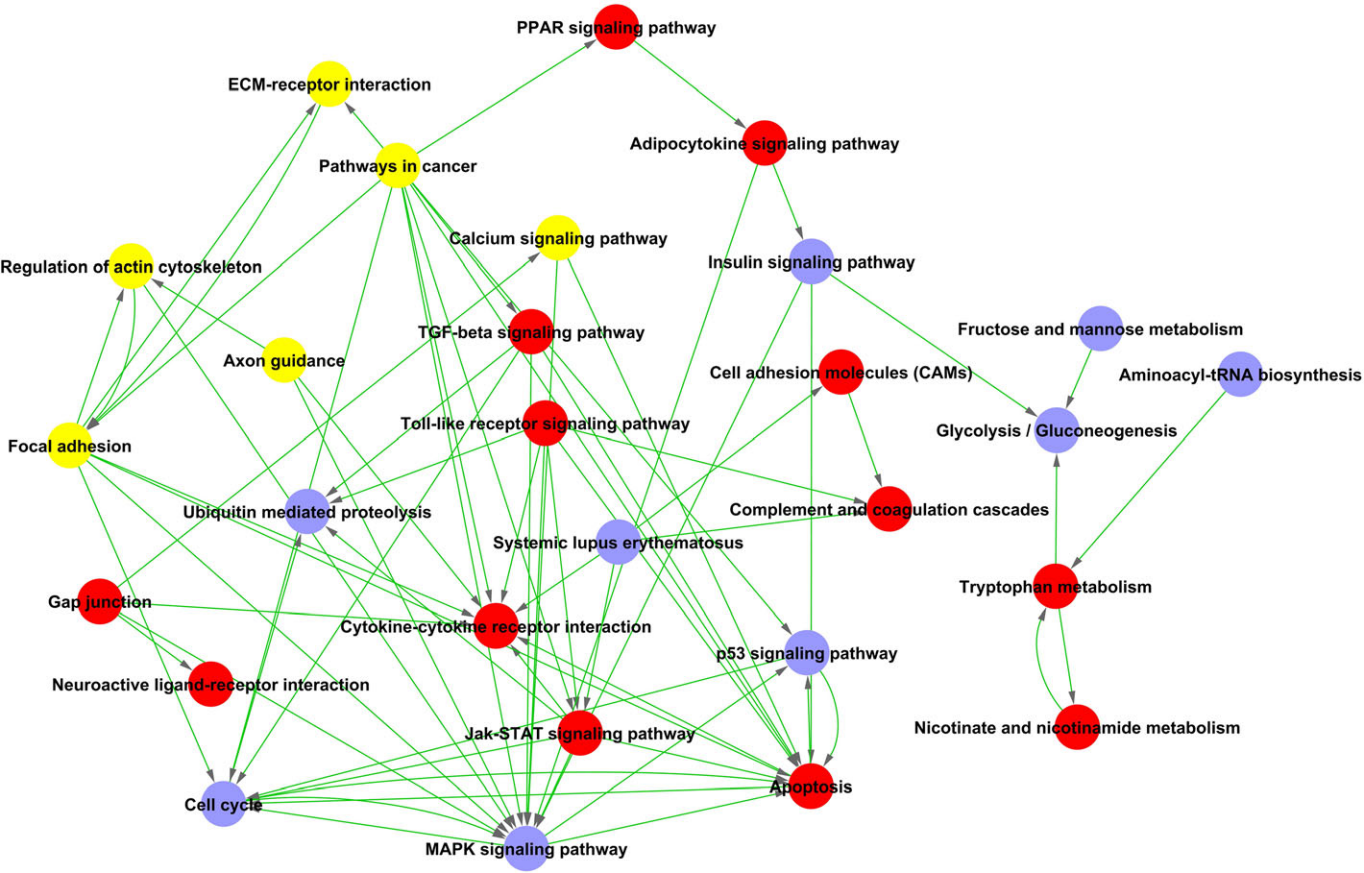
The interaction network of significant pathways (Path-net). The role of each pathway in the network was measured by counting its connections to upstream and downstream pathways. A pathway with a high degree implied that it played an important role in the signaling network. *Blue* represents downregulated pathways, *red* represents upregulated pathways, and *yellow* represents up- and downregulated pathways. The lines indicate interactions between pathways. *ECM* extracellular matrix, *TGF* transforming growth factor

Among the significant pathways related to differential genes, a total of 170 upregulated genes and 152 downregulated genes was identified according to the *p* value (*p* < 0.05; Additional file 5: Table S5). The Path-Net was the interaction net of the significant pathways of the differential expression genes, and was built according to the interaction among pathways of the KEGG database to find the interaction between the significant pathways directly and systemically. After performing Path-net analysis to generate an interaction network of these significantly changed pathways, we could identify the relationship between the pathways enriched from differential expression genes and identify why a certain pathway was activated (Fig. 2). Our results identified the crosstalk of key pathways which may have an important role in the differentiation regulation of BMSCs. The crosstalk among the MAPK signaling pathway, the Jak-STAT signaling pathway, the Toll-like receptor signaling pathway, and the transforming growth factor (TGF)-beta signaling pathway were identified to a high degree (Fig. 2; Additional file 6: Table S6). We observed a high degree of increased activity of apoptosis, the cytokine-cytokine receptor interaction, the TGF-beta signaling pathway, the Jak-STAT signaling pathway, and the Toll-like receptor signaling pathway in a cluster of upregulating genes, and a decreased activity of the MAPK signaling pathway, the cell cycle, and the p53 signaling pathway in a cluster of downregulating genes (Fig. 2; Additional file 6: Table S6).

**Fig. 3:**
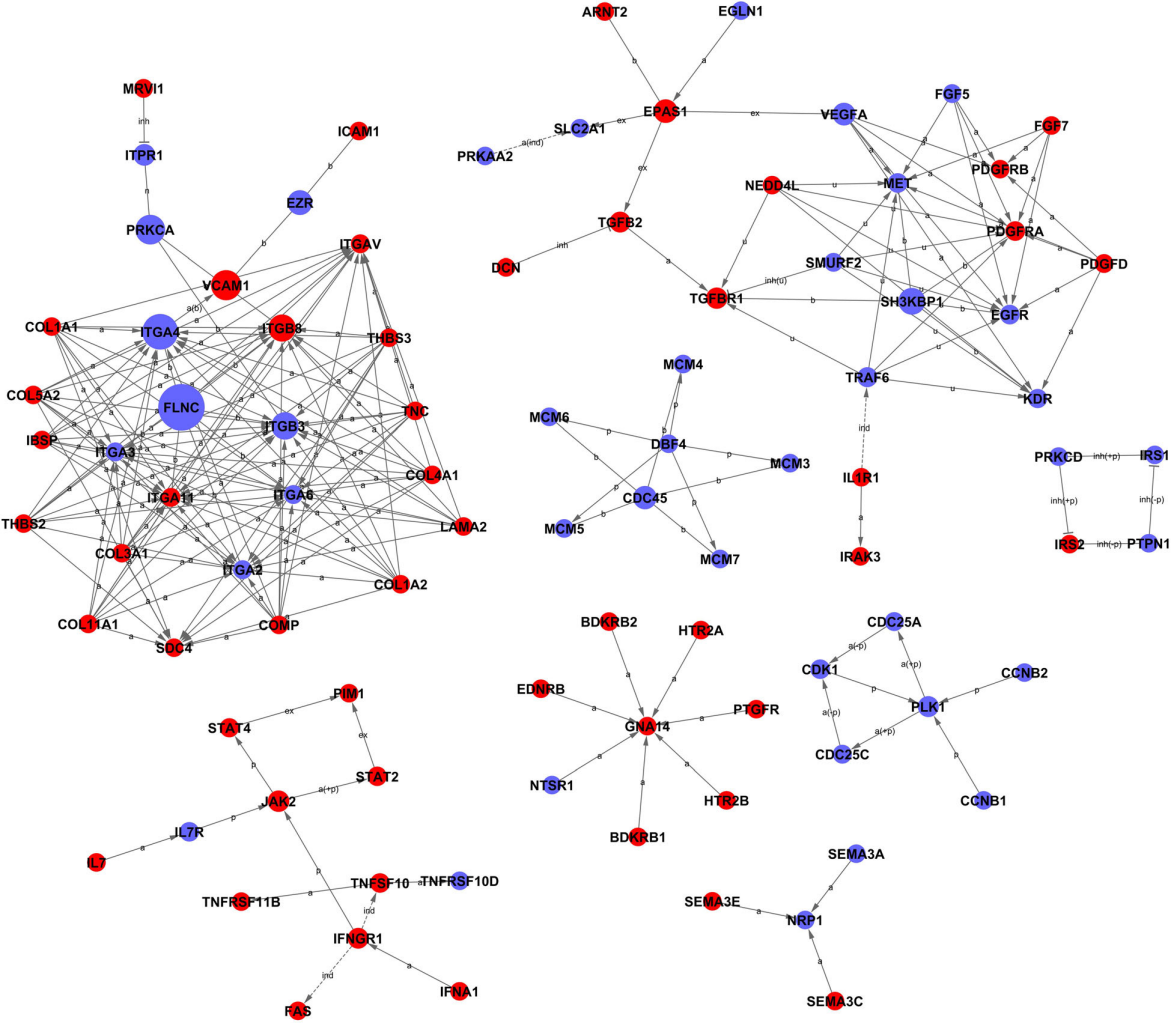
The interaction network of differentially expressed genes (Signal-net). In the Signal-net, the genes are characterized by measuring their “betweenness centrality”, the number of times a node is located in the shortest path between two other nodes. This measure reflects the importance of a node in a network relative to another. The *circles* represent important functional genes (*red*: upregulated genes; *blue*: downregulated genes); the circle size represents the degree of interaction (betweenness centrality), and lines indicate the interactions

We constructed an interactions repository by a Signal-net analysis (Signal-net) based on the significantly regulated GOs and pathways to identify 92 core genes during osteogenic differentiation of BMSCs according to the degree of gene interaction (betweenness centrality) (Fig. 3; Additional file 7: Table S7).

To obtain core mRNAs and lncRNA genes during osteogenic differentiation of MSCs, we calculated the Pearson correlation for each pair of genes and used the most significantly correlated pairs to construct a coding-noncoding gene co-expression (CNC) network to identify the interactions and significance degree amongst the differentially expressed lncRNAs and mRNAs according to clustering coefficient and degree (Additional file 8: Figure S1 and Additional file 9: Figure S2). The closely correlated mRNAs and lncRNAs were identified. One mRNA was correlated with one or more lncRNAs and vice versa. We identified 13 core regulatory genes including seven mRNAs (GPX3, TLR2, BDKRB1, FBXO5, BRCA1, MAP3K8, and SCARB1) and six lncRNAs (XR_111050, NR_024031, FR374455, FR401275, FR406817, and FR148647) according to clustering coefficient and degree value (Additional file 10: Table S8; clustering coefficient ≥0.9 and degree >7).

### The lncRNA XR_111050 enhanced the osteogenic differentiation potentials of MSCs

**Fig. 4:**
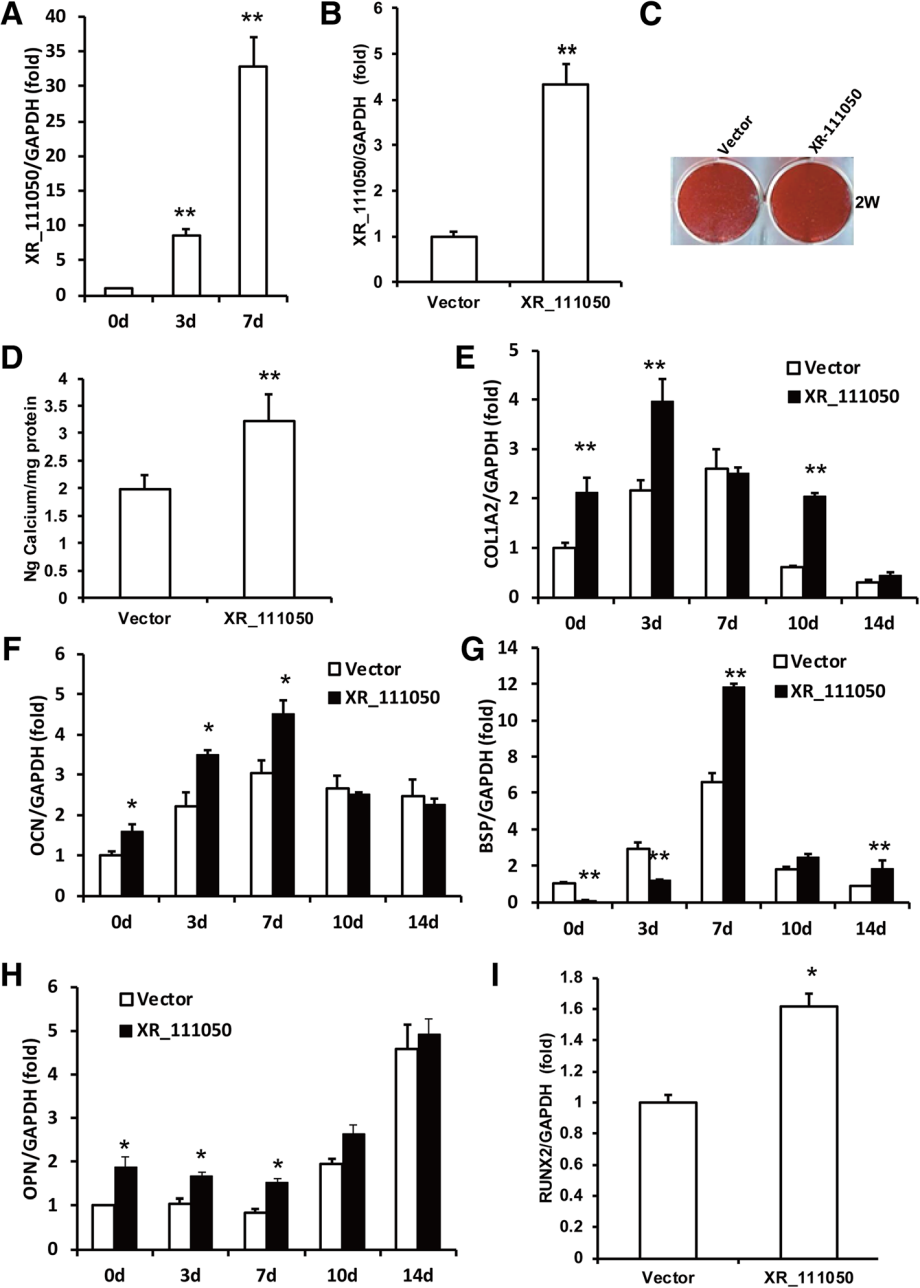
XR_111050 enhanced the osteogenic differentiation of BMSCs. The BMSCs were transduced with XR_111050 lentiviral vector or with empty vector. **a** XR_111050 expression increased during osteogenic differentiation as evaluated by qRT-PCR. **b** The enhanced XR_111050 expression in BMSCs was verified with qRT-PCR. The increased XR_111050 expression enhanced mineralization of BMSCs as shown by **c** Alizarin red staining and **d** calcium quantitative analysis. qRT-PCR results showed that enhanced XR_111050 expression resulted in upregulated expressions of COL1A2 (**e**), OCN (**f**), BSP (**g**) and OPN (**h**) after osteogenic induction. **i** The enhanced XR_111050 expression increased RUNX2 expression in BMSCs. GAPDH was used as an internal control. Student’s *t* test was performed to determine statistical significance. Error bars represent SD (*n* = 3). *p < 0.05, **p < 0.01. *BSP* bone sialoprotein, *COL1A2* collagen a-2(I) chain, *d* days, *GADPH* glyceraldehyde-3-phosphate dehydrogenase, *OPN* osteopontin, *RUNX2* runt-related transcription factor 2, *W* weeks

**Fig. 5:**
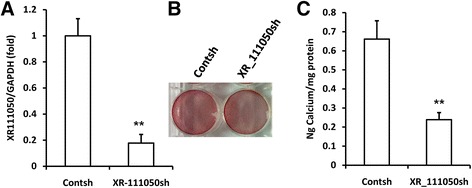
XR_111050 knockdown inhibited osteogenic differentiation of BMSCs. The BMSCs was transduced with short-hairpin RNAs (shRNA) to silence XR_111050 (*XR_111050sh*) or with control shRNA (*Contsh*). **a** The reduced XR_111050 expression by shRNA was evaluated by qRT- PCR. *XR_111050* knockdown inhibited mineralization, as shown by results of Alizarin red staining (**b**) and calcium quantitative analysis (**c**). Error bars represent SD (*n* = 3). **p < 0.01. *GADPH* glyceraldehyde-3-phosphate dehydrogenase

In the CNC networks, among the six lncRNAs, XR_111050 showed the most significant clustering coefficient. The RT-PCR results showed that the expression of XR_111050 increased along with osteogenic differentiation of BMSCs (Fig. 4a). To evaluate the effect of XR_111050 on osteogenesis, we inserted the XR_111050 sequence into a lentiviral vector, which was then transduced into BMSCs via lentiviral infection. Enhanced expression of XR_111050 was verified with RT-PCR in BMSCs (Fig. 4b). Two weeks after osteogenic induction, our results showed that XR_111050 overexpression enhanced osteogenic differentiation of BMSCs as shown by Alizarin red staining and quantitative calcium measurements compared with cells infected with the empty vector (Fig. 4c and d). RT-PCR analysis of XR_111050-overexpressing BMSCs showed stronger expression of osteogenic markers (including COL1A2 at 0, 3, and 10 days, OCN and OPN at 0, 3, and 7 days, and BSP at 7 and 14 days after osteogenic induction) compared with the control group (Fig. 4e–h). We also examined expression of the key transcription factors RUNX2 and OSX for regulating osteogenic differentiation. RT-PCR results showed that XR_111050-overexpressing BMSCs had a significantly higher RUNX2 mRNA level compared with cells infected with the empty vector (Fig. 4i). On the other hand, OSX expression levels did not significantly differ between groups (data not shown). We designed and introduced a short-hairpin RNA (shRNA) to target XR_111050. After selection, the knockdown efficiency (80%) was verified by RT-PCR (Fig. 5a). Two weeks after osteogenic induction, Alizarin red staining and quantitative calcium measurements revealed that mineralization was markedly decreased after depletion of XR_111050 in BMSCs (Fig. 5b and c).

**Fig. 6:**
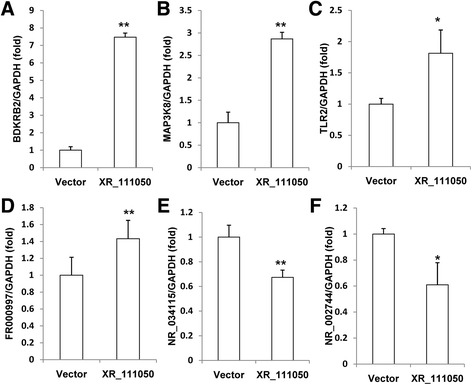
XR_111050 regulated the expression of BDKRB2, MAP3K8, TLR2, FR000997, NR_002744, and NR_034115 in BMSCs. qRT-PCR results showed that enhanced XR_111050 expression resulted in upregulated expression of BDKRB2 (**a**), MAP3K8 (**b**), TLR2 (**c**), and FR000997 (**d**), and downregulated expression of NR_034115 (**e**) and NR_002744 (**f**). GAPDH was used as an internal control. Student’s *t* test was performed to determine statistical significance. Error bars represent SD (*n* = 3). *p < 0.05, **p < 0.01. *GADPH* glyceraldehyde-3-phosphate dehydrogenase

We then detected the expression of BDKRB2, MAP3K8, SCARB1, TLR2, FR000997, FR374455, NR_002744, NR_027293, and NR_034115 in BMSCs after overexpression of XR_111050 to investigate possible correlations. The RT-PCR results showed that overexpression of XR_111050 upregulated the expression of BDKRB2, MAP3K8, TLR2, and FR000997 (Fig. 6a–d), and downregulated the expression of NR_002744 and NR_034115 compared with control group (Fig. 6e and f), but the expression of SCARB1, FR374455, and NR_027293 were not changed (data not shown).

To confirm whether XR_111050 had similar functions in other MSCs, we detected the XR_111050 expression in periodontal ligament stem cells (PDLSCs) and the RT-PCR results showed that the expression of XR_111050 increased along with osteogenic differentiation of PDLSCs (Additional file 11: Figure S3A). Then we overexpressed XR_111050 in PDLSCs via lentiviral infection. Enhanced expression of XR_111050 was verified with RT-PCR in PDLSCs (Additional file 11: Figure S3B). Two weeks after osteogenic induction, Alizarin red staining and quantitative calcium measurements revealed that mineralization was markedly increased after overexpression of XR_111050 in PDLSCs (Additional file 11: Figure S3C,D).

## Discussion

MSC differentiation is precisely regulated and orchestrated by mechanical and molecular signals that have increasingly attracted great attention in recent years [4, 6, 33–38]. Therefore, understanding the molecular mechanisms underlying MSC differentiation and modulating lineage commitment of MSCs is of importance for therapeutic purposes [39].

Intensive studies have demonstrated that a number of critical signaling pathways are involved in regulating the lineage commitment of MSCs, including TGF-β/BMP signaling, Wnt signaling, Hedgehogs, Notch, and FGFs [4, 40–43]. A large range of biological processes have been reported to be associated with lncRNAs, which are correlated with various human diseases and life processes. Emerging evidence is revealing that lncRNAs regulate differentiation of stem cells [44]. Some lncRNAs have been reported to be key regulators of osteogenic differentiation of MSCs in recent studies [27–31, 45, 46]. However, the role of lncRNAs on the lineage commitment of MSCs remains largely unknown.

Here, we organized these studies to outline the profiling of mRNAs and lncRNAs during osteogenic differentiation of BMSCs. By comparing the lncRNA and mRNA expressions of differentiated and undifferentiated BMSCs, we identified 1269 differentially expressed mRNAs and 1408 differentially expressed lncRNAs. Our results provided a dynamic variation profiling in the expressions of lncRNAs and mRNAs during osteogenic differentiation of BMSCs, and implied that upregulated lncRNAs and mRNAs were predominant during osteogenic differentiation of BMSCs. The differentially expressed mRNAs and lncRNAs obtained were used as candidates to screen the key genes controlling osteogenic differentiation of BMSCs by further bioinformatic analysis.

We screened for the important pathways involved in the differentiation mechanism of BMSCs. Among the important pathways, the cytokine-cytokine receptor interaction pathway was demonstrated to be the dominant pathway and 28 genes were enriched. Of these, TNFSF10 was identified as being significantly upregulated, which functions as a negative regulator of bone resorption by activating TNFRSF11B. The interaction or correlation of these pathways were built with Path-Net (the interaction net of the significant pathways of the differential expression genes) according to the interaction amongst pathways of the KEGG database to find the interaction among the significant pathways directly and systemically. A pathway with a high degree implied that it played an important role in the signaling network. The Path-net analysis results indicated that the MAPK signaling pathway, the Jak-STAT signaling pathway, the Toll-like receptor signaling pathway, and the TGF-beta signaling pathway may have important roles in the differentiation regulation of BMSCs to a high degree. The results are consistent with previous reports [4, 41, 47–50]. The Path-net identified in our results also provided insights into the role of crosstalk among key signaling pathways regulating osteogenic differentiation of BMSCs. Integration of signaling into signaling networks through cross-talk with other signal transduction pathways contributes to induction of specific activation and differentiation pathways [51]. We identified that the Toll-like receptor signaling pathway was involved in osteogenic differentiation of BMSCs by targeting apoptosis, cytokine-cytokine receptor interaction, the Jak-STAT signaling pathway, and the MAPK signaling pathway. Recent studies implied that signal transduction crosstalk is regulated in a dynamic manner, and that dysregulation of signal transduction crosstalk may contribute to pathogenesis [52]. For example, our results showed that significantly upregulated IL6 was associated with multiple signaling pathways, including the cytokine-cytokine receptor interaction, the Toll-like receptor signaling pathway, and the Jak-STAT signaling pathway, which can enhance osteogenic differentiation [4, 53].

We then predicted some core regulating factors using bioinformatic analysis of differentially expressed mRNAs and lncRNAs. We constructed a potential interaction between mRNAs and lncRNAs by CNC network, and identified key genes, such as GPX3, TLR2, BDKRB1, MAP3K8, and SCARB1, and lncRNAs, such as XR_111050 and NR_024031. TLR2 and MAP3K8, as component of the Toll-like receptor and MAPK signaling pathways which is upregulated during osteogenic differentiation of BMSCs in this study, may participate in osteogenic differentiation via positive or negative regulation of lncRNAs. Studies have suggested that the osteogenic differentiation seems to be enhanced by TLR2 activation in human BMSCs [50, 54, 55], which may work through NF-κB activation [55, 56], while MAP3K8 is an important mediator of intracellular mechanotransduction in human MSCs which is related to shear stress that can induce osteogenic differentiation of human MSCs [57, 58]. These data may provide clues to the potential role of interaction between lncRNAs and TLR signaling or MAPK signaling on osteogenic differentiation of BMSCs. GPX3, BDKRB1, and SCARB1 are involved in bone formation and absorption [59–61]. GPX3 belongs to the glutathione peroxidase family, which catalyzes the reduction of organic hydroperoxides and hydrogen peroxide by glutathione, and thereby protect cells against oxidative damage [62]. A previous investigation discovered that elderly osteoporotic women with hip fractures had increased expression of GPX3, suggesting increased GPX3 antioxidative activity in bone samples [59]. Bdkrb1 plays an important role in inflammation and healing; Bdkrb1 knockout mice displayed increased bone loss, and bone marrow cells from Bdkrb1 knockout mice exhibited enhanced differentiation into osteoclasts [60]. The Scarb1 gene product is a high-density lipoprotein receptor which was shown to influence bone metabolism; Scarb1 deficiency promoted osteoblastogenesis but stunted terminal osteocyte differentiation [61]. When studying NR_024031, also named DANCR, researchers found that DANCR promoted the expression of IL-6 and TNF-α, and DANCR-induced IL-6 and TNF-α had bone-resorbing activity indicating that DANCR is involved in the pathology of osteoporosis and may be a biomarker for postmenopausal osteoporosis [63]. DANCR can inhibit the Wnt/β-catenin signal pathway and suppress the odontogenic differentiation in human dental pulp cells [64]. Moreover, DANCR can be activated by Sox4 and promotes cell proliferation and chondrogenesis in synovium-derived MSCs [65]. These reports suggest that GPX3, BDKRB1, SCARB1, and DANCR may play important roles in the osteogenic differentiation of BMSCs.

In the present study, we selected one candidate lncRNA, XR_111050, to investigate its function on the osteogenic differentiation of BMSCs. Our experiments showed that XR_111050 resulted in increased mineralization in BMSCs. Upregulated osteogenic marker (COL1A2, OCN, BSP, and OPN) mRNA levels verified that XR_111050 enhanced in vitro osteogenic differentiation in BMSCs. The activation of multiple transcription factors, such as RUNX2 and OSX, is associated with MSC osteogenic differentiation [66]. Our study found that XR_111050 stimulated the expression of one key transcription factor, RUNX2, but not the expression of another, OSX, which is involved in osteogenic differentiation. In addition, our results also confirmed that XR_111050 could enhance mineralization in PDLSCs. Taken together, our findings identified that XR_111050 was an enhancer of the osteogenic differentiation regulation of MSCs. XR_111050 is predicted to be a human hypothetical LOC100509635 miscRNA, a general term for a series of miscellaneous small RNA, which serves a variety of functions, including some enzyme-like catalysis and RNA processing [67]. Previous studies demonstrated that lncRNAs may function by controlling the transcriptional regulation of neighboring coding genes in a *cis* or *trans* manner [16]. Identifying differentially expressed nearby coding mRNA may enhance our understanding of the function of lncRNAs on the BMSCs. In this study, we found that XR_111050 is closely correlated with BDKRB2, MAP3K8, SCARB1, TLR2, FR000997, FR374455, NR_002744, NR_027293, and NR_034115 by lncRNA–mRNA CNC network analysis. We have detected their expression in BMSCs after overexpression of XR_111050 to investigate their correlations and possible mechanism. The results showed that overexpression of XR_111050 enhanced the expression of BDKRB2, MAP3K8, TLR2, and FR000997, and inhibited the expression of NR_002744 and NR_034115. Our microarray data showed that the expression of BDKRB2, MAP3K8, TLR2, and FR000997 was increased, and that of NR_002744 and NR_034115 was decreased after osteogenic differentiation in BMSCs, consistent with the results of overexpression of XR_111050, indicating that BDKRB2, MAP3K8, TLR2, and FR000997 might be positive regulators and NR_002744 and NR_034115 might be negative regulators for osteogenic differentiation of BMSCs. In conclusion, these results implied that XR_111050 may play a role in the osteogenic differentiation regulation of MSCs via a large number of cooperators or downstream targets, such as DKRB2, MAP3K8, TLR2, FR000997, NR_002744, and NR_034115. However, further studies must be performed to investigate this hypothesis.

## Conclusions

We identified the differential expression profiling of coding and long noncoding transcripts during osteogenic differentiation of BMSCs with human whole transcriptome microarray. The potential regulatory mechanisms were identified with bioinformatic analyses. We further predicted the interactions of differentially expressed coding and noncoding genes, and identified core regulatory factors by co-expression networks during osteogenic differentiation of BMSCs. Our results also suggested that one lncRNA, XR_111050, is required for osteogenic differentiation of MSCs. Our studies provide a base for further understanding the precise role and mechanisms of these lncRNAs and mRNAs controlling osteogenic differentiation of MSCs and provide an important resource for investigation of MSC biology. In addition, our obtained lncRNAs with differential expression during MSC osteogenic differentiation could be potential targets for bone tissue engineering.

## Methods

### Cell culture and osteogenic differentiation

BMSCs derived from 18- to 20-year-old males (*n* = 3) were obtained from Cyagen Biosciences (Guangzhou, China). Human impacted third molars were collected from healthy male patients (16–20 years old). Teeth were first disinfected with 75% ethanol and then washed with phosphate-buffered saline. PDLSCs were isolated, cultured, and identified as previously described [68]. Briefly, PDLSCs were separated from periodontal ligament in the middle one-third of the root. Subsequently, the periodontal ligament tissue was digested in a solution of 3 mg/mL collagenase type I (Worthington Biochemical Corp., Lakewood, NJ, USA) and 4 mg/mL dispase (Roche Diagnostics Corp., Indianapolis, IN, USA) for 1 h at 37 °C. Single-cell suspensions were obtained by cell passage through a 70-μm strainer (Falcon, BD Labware, Franklin Lakes, NJ, USA). MSC cultures were grown in a humidified, 5% CO_2_ incubator at 37 °C in DMEM alpha modified Eagle’s medium (Invitrogen, Carlsbad, CA, USA), supplemented with 15% fetal bovine serum (FBS; Invitrogen, Carlsbad, CA, USA), 2 mmol/L glutamine, 100 U/mL penicillin, and 100 μg/mL streptomycin (Invitrogen, Carlsbad, CA, USA). The culture medium was changed every 3 days. All MSCs were used in subsequent experiments after 3–5 passages. For osteogenesis differentiation, MSCs were seeded at a density of 2.0 × 10^5^ cells/well into six-well plates with routine medium. When cells reached 80–90% confluence, the medium was changed, and cells were grown in osteogenic differentiation medium with the STEMPRO osteogenesis differentiation Kit (Invitrogen). The osteogenic differentiation medium was replaced every 3 days.

### Total RNA isolation and microarray hybridization

BMSCs were grown in osteogenic differentiation medium for 7 days using the StemPro osteogenesis differentiation kit (Invitrogen); the osteogenic differentiation medium was changed every 3 days. Three total RNA samples from BMSCs of three different individuals were extracted separately using TRIzol and the RNeasy mini kit (Qiagen, Germany). RNA quality and quantity were confirmed by multiImager and spectrophotometer (Meriton, China). Each total RNA sample was further purified using the RNeasy mini kit and RNase-Free DNase Set (QIAGEN, GmBH, Germany).

Microarray analysis was performed using Human Transcriptome Array (Affymetrix), covering more than 285,000 coding and noncoding transcripts (>245,000 coding transcripts, >40,000 noncoding transcripts). For each experimental group, three biological replicates (three BMSCs RNA samples from three different individuals) were hybridized. The RNA labeling and microarray hybridization were carried out according to the Affymetrix expression analysis technical manual (by Genminix Informatics Ltd., Shanghai, China). The arrays were scanned using the GeneChip® scanner 3000 (Affymetrix, CA, USA). The microarray raw data were normalized with Affymetrix Expression Console software using the MAS5 statistical algorithm.

### Quantitative RT-PCR

qRT-PCR was used to verify the differential expression of genes that were detected on the microarray and to detect the expression of osteogenic differentiation markers. At days 0, 3, 7, 10, and 14 after osteogenic induction, BMSCs were harvested for RNA extraction. Total RNA was isolated from BMSCs with Trizol reagents (Invitrogen). For qRT-PCR, 2 μg aliquots of RNA as a template were combined with random hexamers and reverse transcriptase, according to the manufacturer’s protocol (Invitrogen). qRT-PCR was performed using QuantiTect SYBR Green PCR kit (Qiagen, Germany) and an iCycler iQ Multicolor Real-Time PCR Detection System. Primer sequences used for qRT-PCR are listed in Additional file 12 (Table S9). Relative mRNA levels were calculated using the 2^−ΔΔCt^ method. The ΔCts were obtained from Ct normalized with GAPDH. Pearson’s correlation coefficient was further calculated for each gene using the normalized data to quantify the consistency between microarray experiments and qRT-PCR (*p* < 0.05 and *R* > 0.9).

### Bioinformatic analysis

The bioinformatic analysis was performed (by Genminix Informatics Ltd., Shanghai, China) as described previously [68]. Briefly, after normalized data were compared and filtered, the differentially expressed genes for the control and experiment group were firstly filtered using RVM *t* test (Two Class Dif) (*p* < 0.05) for following analysis. Hierarchical clusters were performed by EPCLUST.

GO analysis was applied to analyze the main function of the differential expression genes according to the GO using the two-sided Fisher’s exact test and *χ* ^2^ test for classifying the GO category (*p* < 0.05, FDR < 0.01). Enrichment of the GO category was calculated for significance of the function. Go-map analysis was made to identify the interaction net of the significant GOs of the differential expression genes.

Pathway analysis was used to discover the significant pathway of the differential genes according to KEGG, Biocarta and Reatome, using Fisher’s exact test and *χ* ^2^ test (*p* < 0.05, FDR < 0.01). The enrichment Re of pathways was calculated. The interaction net of the significant pathways of the differential expression genes (Path-Net) was built to find the interaction among the significant pathways directly and systemically according to the interaction among pathways of the KEGG database.

The gene-gene interaction network (Signal-Net) from differentially expressed genes was constructed to identify the molecular networks between the two genes based on the interaction database from KEGG.

We built a coding-noncoding gene co-expression network (CNC) to further identify the interactions and locate core regulatory factors (genes) among genes according to the normalized signal intensity of specific expression genes.

### Plasmid construction and viral infection

The plasmids were constructed with standard methods; all structures were verified by appropriate restriction digestion and/or sequencing. Human full-length XR-111050 cDNA from BMSCs was produced with a standard PCR protocol. This sequence was subcloned into the LV5 lentiviral vector (Genepharma Company, Suzhou, China). Short-hairpin RNAs (shRNA) with the complementary sequences of the target genes were subcloned into the LV3 lentiviral vector (Genepharma Company, Suzhou, China). For viral infections, MSCs were plated overnight and then infected with lentiviruses in the presence of polybrene (6 μg/mL; Sigma-Aldrich, St. Louis, MO, USA) for 6 h. After 48 h, infected cells were selected with 1 μg/mL puromycin for 7 days. The target sequences for the shRNA were: LV3 shRNA (Consh), 5’-TTCTCCGAACGTGTCACGTTTC-3’; XR-111050 shRNA (XR-111050sh), 5’-GGACGTGTCTTTCAGGGAAAG-3’.

### Alizarin red detection and quantitative calcium analysis

MSCs were grown in osteogenic differentiation medium using the StemPro osteogenesis differentiation kit (Invitrogen). For detecting mineralization, cells were induced for 2 weeks, fixed with 70% ethanol, and stained with 2% Alizarin red (Sigma-Aldrich, St. Louis, MO, USA). To quantitatively determine calcium, Alizarin red was destained with 10% cetylpyridinium chloride in 10 mM sodium phosphate for 30 min at room temperature. The concentration was determined by measuring the absorbance at 562 nm on a multiplate reader and comparing to a standard calcium curve with calcium dilutions in the same solution. The final calcium level in each group was normalized to the total protein concentration detected in a duplicate plate.

### Statistics

All statistical calculations were performed with SPSS11 statistical software. Statistical analyses included comparisons with the *t* test, Fisher’s exact test, $\chi$ ^2^ test, and the Pearson correlation, as appropriate; *p* values less than 0.05 were considered statistically significant.

## Additional files

Additional file 1: Table S1. The differentially expressed mRNAs during osteogenic differentiation of BMSCs. (XLS 139 kb)
Additional file 2: Table S2. The differentially expressed lncRNAs during osteogenic differentiation of BMSCs. (XLS 185 kb)
Additional file 3: Table S3. The upregulated and downregulated GO functions during osteogenic differentiation of BMSCs. (XLS 54 kb)
Additional file 4: Table S4. The upregulated and downregulated pathways during osteogenic differentiation of BMSCs. (XLS 33 kb)
Additional file 5: Table S5. The upregulated and downregulated genes in significant pathways during osteogenic differentiation of BMSCs. (XLS 111 kb)
Additional file 6: Table S6. The significant pathways enriched by Path-net during osteogenic differentiation of BMSCs. (XLS 25 kb)
Additional file 7: Table S7. The 92 core genes enriched by Signal-net during osteogenic differentiation of BMSCs. (XLS 37 kb)
Additional file 8: Figure S1. The co-expression network of mRNAs and lncRNAs in BMSCs before osteogenic differentiation (CNC). Circles represent upregulated (red) genes, and downregulated (blue) genes in BMSCs. The lncRNA genes are encircled in yellow. The lines represent the regulatory relationships between genes (solid lines represent positive correlations, dotted lines represent negative correlations). The circle size represents the degree of centrality. (PNG 3798 kb)
Additional file 9: Figure S2. The co-expression network of mRNAs and lncRNAs in BMSCs after osteogenic differentiation (CNC). Circles represent upregulated (red) genes, and downregulated (blue) genes in BMSCs. The lncRNA genes are encircled in yellow. The lines represent the regulatory relationships between genes (solid lines represent positive correlations, dotted lines represent negative correlations). The circle size represents the degree of centrality. (PNG 4510 kb)
Additional file 10: Table S8. The 13 core regulatory genes by CNC during osteogenic differentiation of BMSCs. (XLS 27 kb)
Additional file 11: Figure S3. XR_111050 enhanced the osteogenic differentiation of PDLSCs. The PDLSCs were transduced with XR_111050 lentiviral vector or with empty vector. (A) XR_111050 expression increased during osteogenic differentiation evaluated by qRT-PCR. (B) The enhanced XR_111050 expression in PDLSCs was verified with qRT-PCR. (C,D) The enhanced XR_111050 expression enhanced mineralization of PDLSCs shown by Alizarin red staining (C) and calcium quantitative analysis (D). Error bars represent SD (*n* = 3). ***p* < 0.01. (TIFF 1312 kb)
Additional file 12: Table S9. The primer sequences used for qRT-PCR. (XLS 30 kb)

## Abbreviations

BMSC: Bone marrow mesenchymal stem cell
CNC: Coding-noncoding gene co-expression
ECM: Extracellular matrix
FDR: False discovery rate
GO: Gene ontology
lncRNA: Long noncoding RNA
MSC: Mesenchymal stem cell
Path-net: Interaction network of significant pathways
PDLSC: Periodontal ligament stem cell
(q)RT- PCR: (Quantitative) real-time polymerase chain reaction
Signal-net: Gene-gene interaction network
TGF: Transforming growth factor
