# Supplementary figures and images for: Differential long noncoding RNA/mRNA expression profiling and functional network analysis during osteogenic differentiation of human bone marrow mesenchymal stem cells
Source: Stem Cell Res Ther. 2017 Feb 7;8:30. doi: 10.1186/s13287-017-0485-6 (PMC5297123; doi:10.1186/s13287-017-0485-6)

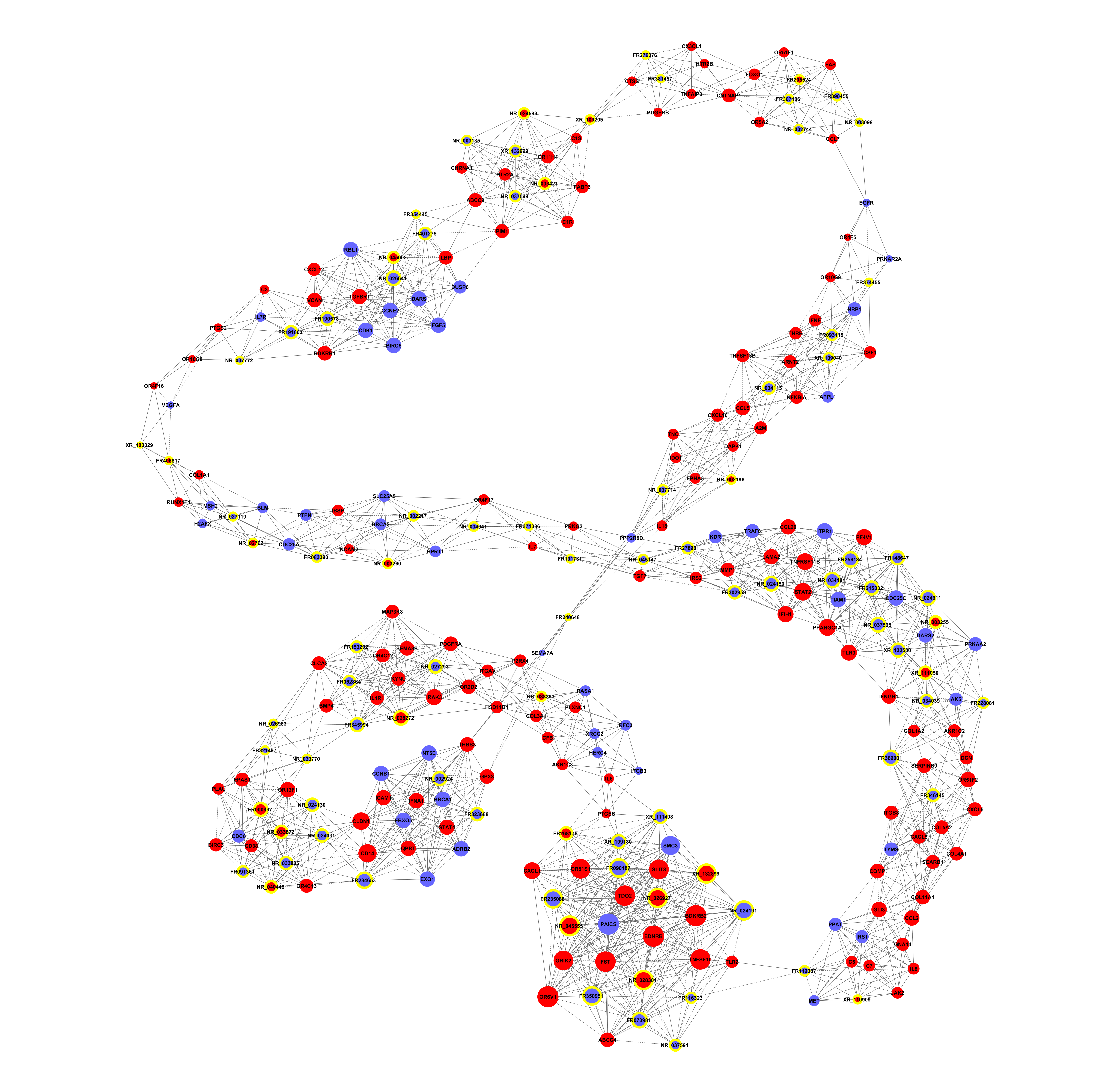

Supplement: Additional file 8: Figure S1. — The co-expression network of mRNAs and lncRNAs in BMSCs before osteogenic differentiation (CNC). Circles represent upregulated (red) genes, and downregulated (blue) genes in BMSCs. The lncRNA genes are encircled in yellow. The lines represent the regulatory relationships between genes (solid lines represent positive correlations, dotted lines represent negative correlations). The circle size represents the degree of centrality. (PNG 3798 kb) [file 13287_2017_485_MOESM8_ESM.png]

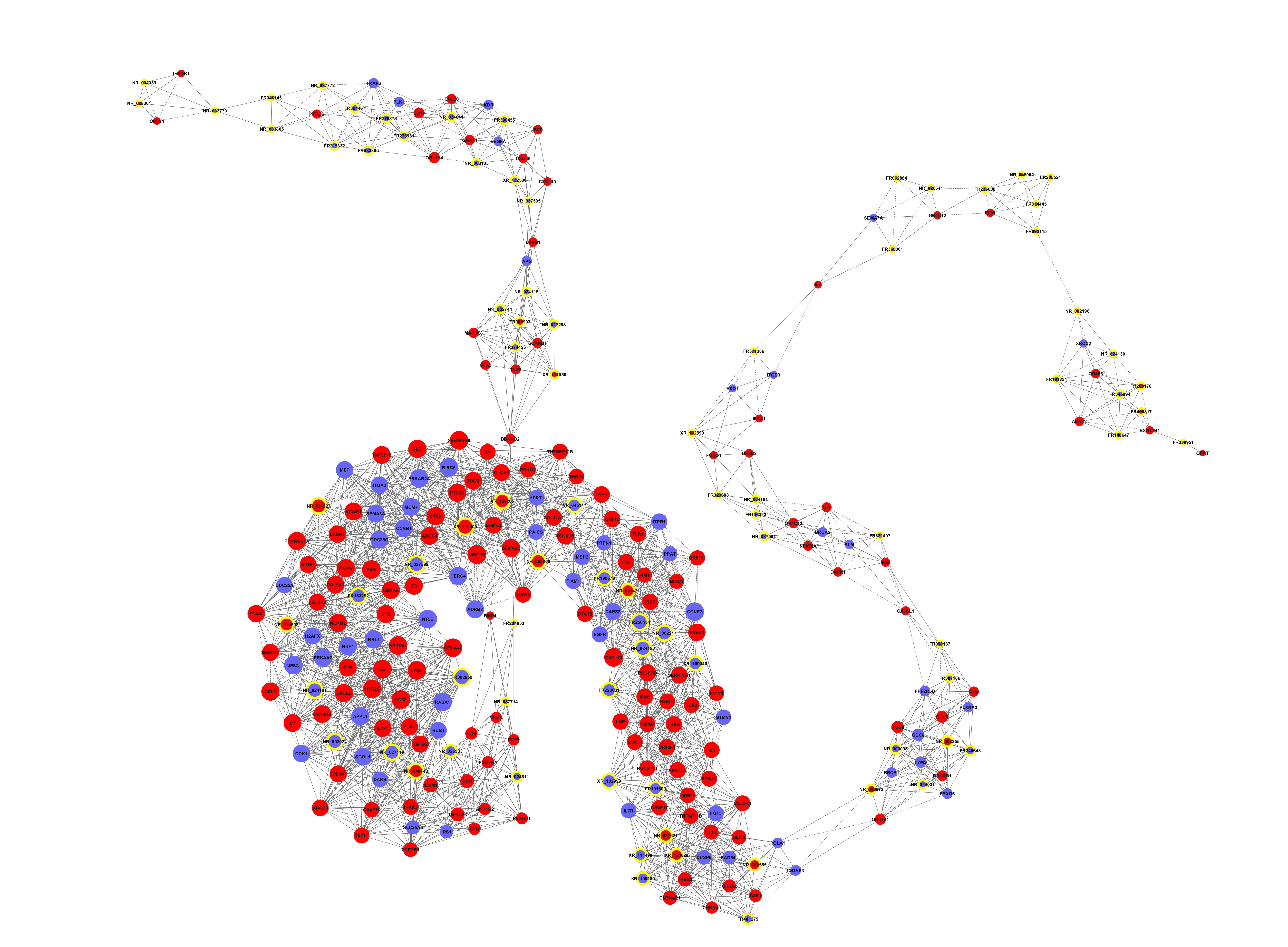

Supplement: Additional file 9: Figure S2. — The co-expression network of mRNAs and lncRNAs in BMSCs after osteogenic differentiation (CNC). Circles represent upregulated (red) genes, and downregulated (blue) genes in BMSCs. The lncRNA genes are encircled in yellow. The lines represent the regulatory relationships between genes (solid lines represent positive correlations, dotted lines represent negative correlations). The circle size represents the degree of centrality. (PNG 4510 kb) [file 13287_2017_485_MOESM9_ESM.png]

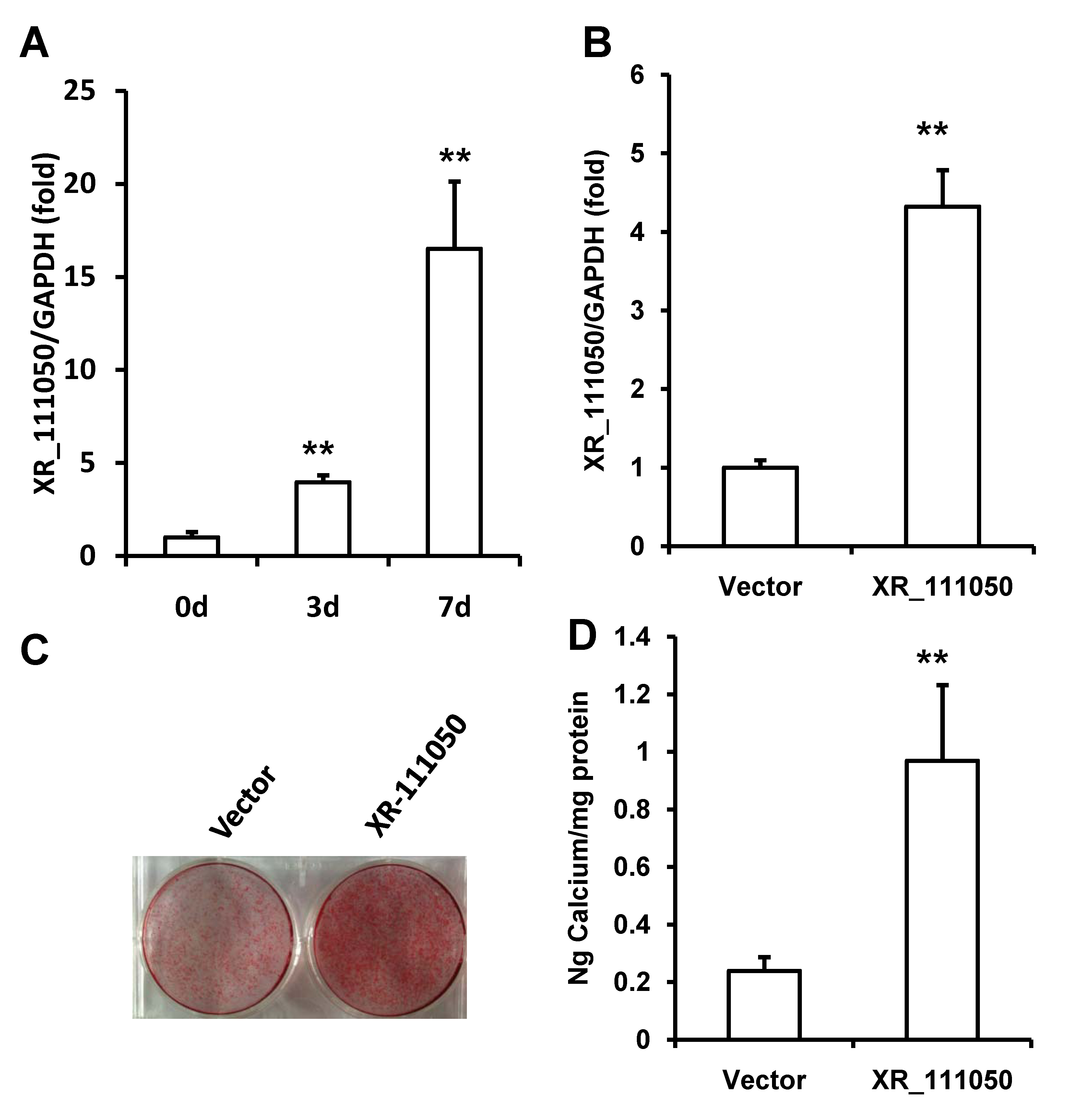

Supplement: Additional file 11: Figure S3. — XR_111050 enhanced the osteogenic differentiation of PDLSCs. The PDLSCs were transduced with XR_111050 lentiviral vector or with empty vector. (A) XR_111050 expression increased during osteogenic differentiation evaluated by qRT-PCR. (B) The enhanced XR_111050 expression in PDLSCs was verified with qRT-PCR. (C,D) The enhanced XR_111050 expression enhanced mineralization of PDLSCs shown by Alizarin red staining (C) and calcium quantitative analysis (D). Error bars represent SD (n = 3). **p < 0.01. (TIFF 1312 kb) [file 13287_2017_485_MOESM11_ESM.tiff]
